# FGF2 Deficiency Modulates Early Microglial Responses Without Affecting Photoreceptor Survival in a Retinitis Pigmentosa Mouse Model

**DOI:** 10.3390/cells15070643

**Published:** 2026-04-02

**Authors:** Felia C. Haffelder, Nundehui Díaz-Lezama, Zeynep Okutan, Claudia Grothe, Susanne F. Koch

**Affiliations:** 1 Department of Pharmacy, Center for Drug Research, Ludwig-Maximilians-Universität München, 81377 Munich, Germany; felia.haffelder@cup.uni-muenchen.de (F.C.H.); nundehui.diaz-lezama@cup.uni-muenchen.de (N.D.-L.); zeynep.okutan@cup.uni-muenchen.de (Z.O.); 2 Institute of Neuroanatomy and Cell Biology, Hannover Medical School, 30625 Hannover, Germany

**Keywords:** fibroblast growth factor 2 (FGF2), photoreceptor degeneration, microglia, retinitis pigmentosa, neuroinflammation, retinal pigment epithelium (RPE)

## Abstract

**Highlights:**

**What are the main findings?**

- FGF2 is dispensable for maintaining retinal architecture, photoreceptor integrity, Müller glia morphology, and vascular organization.
- FGF2 loss does not accelerate photoreceptor degeneration in a murine retinitis pigmentosa model, but microglia numbers were altered at early disease stages.

**What are the implications of the main findings?**

- FGF2 is unlikely to be essential for structural maintenance of the retina or for preventing photoreceptor degeneration in this retinitis pigmentosa model.
- FGF2 may play a role in regulating early microglial responses during retinal degeneration.

**Abstract:**

Fibroblast growth factor 2 (FGF2) is expressed in retinal Müller glia cells, and its expression increases in response to photoreceptor degeneration. To investigate the physiological relevance of FGF2, we analyzed retinal morphology and cellular responses in *Fgf2*-deficient (*Fgf2*^−/−^) mice. Loss of FGF2 did not affect photoreceptor survival, retinal vasculature, or retinal pigment epithelium (RPE) integrity. To further understand its role in retinal degeneration, *Fgf2*^−/−^ mice were crossed with *Pde6b^STOP/STOP^* mice, a model of retinitis pigmentosa (RP). We then analyzed outer nuclear layer thickness, cone number, rod outer segments length, RPE morphology, and microglia number in *Fgf2*^−/−^ *Pde6b^STOP/STOP^* and *Pde6b^STOP/STOP^* mice. Although FGF2 was upregulated in degenerating photoreceptor cells in the *Pde6b^STOP/STOP^* retina, its absence did not accelerate photoreceptor loss in *Fgf2*^−/−^ *Pde6b^STOP/STOP^* mice. Interestingly, microglia numbers were significantly changed at early disease stages in *Fgf2*^−/−^ *Pde6b^STOP/STOP^* retinas compared with *Pde6b^STOP/STOP^* controls, suggesting that FGF2 modulates inflammatory signaling. Together, these results show that loss of FGF2 does not alter photoreceptor degeneration kinetics or retinal morphology, but may contribute to the regulation of early microglial accumulation during degeneration.

## 1. Introduction

FGF2 is a multifunctional member of the FGF family with well-established roles in cell survival, angiogenesis, and tissue repair [1,2,3,4,5]. In the healthy retina, endogenous FGF2 expression is low and largely restricted to the soma of Müller glial cells [6]. Müller glia, the principal macroglial cells of the retina, span the entire thickness of the neural retina and envelop all retinal neurons. They are essential for maintaining retinal health and functionality. For example, they provide metabolic support to photoreceptors, recycle neurotransmitters, and act as optical fibers. In response to photoreceptor degeneration, Müller cells release cytokines, further influencing immune activity, including that of microglia. Microglial cells, the macrophages of the retina, play a pivotal role in retinal immune responses. Müller glia may also contribute to guiding microglial migration toward sites of degeneration. The close spatial and functional associations of microglia with Müller glia processes in the plexiform layers of the retina underscore the critical role of their interaction in maintaining retinal immune homeostasis and in effective immune responses [7].

FGF2 is strongly upregulated in response to retinal stress, following laser-induced injury [8], light damage [9,10,11], hypoxia [12], and inherited retinal degeneration [11,13]. In many of these cases, increased FGF2 expression has been associated with protective effects on retinal neurons [10,12]. In vitro studies have demonstrated that FGF2 stimulates the differentiation and survival of purified photoreceptors from newborn rats [14,15] and the survival of adult pig photoreceptor cells [16]. Consistent with these findings, in vivo studies in animal models of photoreceptor degeneration suggest that FGF2 promotes photoreceptor survival. Injection of FGF2 into the eye, intravitreally or subretinally, delayed photoreceptor degeneration in the Royal College of Surgeons (RCS) rat, a model of inherited retinal dystrophy [17], and in a rat model of light damage [18,19]. Subretinal injection of adeno-associated virus (AAV)-mediated transfer of FGF2 to a rat model of retinitis pigmentosa (TgN S334ter-4 rhodopsin) also slowed photoreceptor degeneration [20]. Beyond its neuroprotective effects on photoreceptors, exogenous FGF2 has also been shown to influence glial plasticity, as treatment with FGF2 in combination with insulin stimulated Müller glia to reprogram into proliferating Müller glia-derived progenitor cells in injured retinas [21]. On the other hand, intravitreal injection of FGF2 into a mouse model of light damage did not rescue photoreceptor cells [22]. Many of these studies rely on pharmacological manipulation to elevate FGF2 levels above physiological concentrations. While these experiments demonstrate the potential neuroprotective capacity of FGF2, they do not clarify the physiological role of endogenous FGF2 in the retina. Recent evidence shows that FGF2 is released after microglial cell death and influences ganglion cell remodeling after ischemic insults in the retina [23]. In addition, FGF2-dependent signaling has been implicated in endothelial metabolic reprogramming and pathological neovascularization in retinal vascular diseases [24].

Thus, despite consistent upregulation of FGF2 during retinal degeneration, its precise functional significance in inherited retinal degeneration remains elusive. In particular, it is unclear whether FGF2 directly modifies disease progression by mediating neuroprotection or whether its upregulation during degeneration reflects a secondary stress response with primary effects on non-neuronal retinal compartments.

To test whether endogenous FGF2 is required for retinal homeostasis and/or modifies the progression of photoreceptor degeneration, we analyzed FGF2 deficiency in healthy and diseased retinas. Using *Fgf2*^−/−^ mice, we assessed the requirement of FGF2 for retinal integrity. In addition, we crossed *Fgf2*^−/−^ mice with the *Pde6b^STOP/STOP^* model of retinitis pigmentosa and examined whether the loss of FGF2 alters photoreceptor degeneration kinetics. By analyzing photoreceptor survival, retinal pigment epithelium (RPE) morphology, microglial activation, and retinal vascular architecture, our study delineates the contribution of endogenous FGF2 to retinal health and inherited retinal degeneration.

## 2. Materials and Methods

### 2.1. Animals

All experiments were performed with adult animals from both sexes. Mice were housed under standard conditions with a 12 h light/dark cycle and ad libitum access to food and water. *Pde6b^STOP/STOP^* mice were generated in the Barbara & Donald Jonas Stem Cell Laboratory, Columbia University, USA [25,26,27]. *Fgf2*^−/−^ mice were kindly provided by Claudia Grothe and carried a neomycin cassette disrupting exon 1 [28,29].

### 2.2. Immunohistochemistry

Retinal cryosections (from 8- and 52-week old mice) were immunolabeled in 5% ChemiBlocker (Merck, Darmstadt, Germany #2170) and 0.3% Triton-X-100 in PBS (pH 7.4) overnight at 4 °C, with primary antibodies against cone arrestin (1:500; Merck, Darmstadt, Germany #AB15282), glutamic-acid-rich protein (GARP; 1:400; Sigma, Burlington, MA, USA #MABN2429), FGF2 (1:25; Merck, Darmstadt, Germany #05-118), CD68 (1:500, Biorad, Hercules, CA, USA #MCA1957T), VEGFA (1:500, CUSABIO, Houston, Texas, USA #CSB-PA08249A0) and glutamine synthetase (1:2000, Abcam, Cambridge, UK #ab228590). Retinal and RPE–choroid–sclera flatmounts were immunolabeled in 5% ChemiBlocker, 3% DMSO, and 0.3% Triton-X-100 in PBS (pH 7.4) overnight at 4 °C, with primary antibodies against Iba1 (1:500; Fujifilm Wako Pure Chemical Corporation, Osaka, Japan #WAKO019-19741) and β-catenin (1:500; Cell Signaling, Danvers, MA, USA #8480), ZO-1 (1:500; Merck, Darmstadt, Germany #MABT11), as well as isolectin GS-IB4 conjugated to fluorescein (1:100; Sigma, Burlington, MA, USA #L2895). Secondary antibodies included anti-rabbit AF647 (1:500; Thermo Fisher Scientific, Waltham, MA, USA #A-21245), anti-rat AF488 (1:500, Thermo Fisher Scientific, Waltham, MA, USA #A-21206), and anti-mouse AF555 (1:500; Thermo Fisher, Waltham, MA, USA #A-31570), applied in 3% ChemiBlocker/PBS (pH 7.4) for 1.5 h at room temperature. Nuclei were counterstained with 5 µg/mL Hoechst 33342 (Invitrogen, Waltham, MA, USA #H1399) for 15 min at room temperature. Sections were mounted in Aqua-Poly/Mount (Polysciences, Warrington, PA, USA #18606) on slides (Thermo Scientific SuperFrost Plus, Thermo Fisher Scientific, Waltham, MA, USA), blinded by assigning them a 5-digit number, and stored at 4 °C.

### 2.3. Imaging and Quantification

Fluorescence images for quantification were acquired with a KEYENCE BZ-X800 microscope (KEYNECE, Oaka, Japan). Measurements of outer nuclear layer (ONL) thickness, rod outer segment (OS) length, and cone number of 8-week and 52-week-old mice were performed using Fiji software (v 2.14.0). OS length was measured 250 μm from the optic nerve head on the ventral side. The cone number was counted in the region of 200–300 µm distance from the optic nerve. ONL thickness was assessed at 250, 500, 750, and 1000 μm from the optic nerve in both ventral and dorsal retina, and spider plots were generated. For microglia quantification (8-week- and 30-week-old mice), 40× confocal Z-stacks were processed, and Iba1-positive cells were quantified and normalized to 1 mm^2^. To quantify the microglial-occupied area in the outer plexiform layer (OPL) of 8-week-old mice, confocal Z-stacks were used as well. Briefly, the z-stack projections were segmented, and a polygon was manually drawn to delimit the microglia’s occupied area. At least 10–12 cells per retinal flatmount were analyzed and averaged (3 mice per group). The number of activated microglia in retinas of 8-week-old mice was quantified in retinal cross-sections based on the colocalization of Iba1 and CD68. To investigate retinal vascular plexiform layers (20-week-old mice), 40× Z-stacks were processed, containing the superficial as well as the stacked intermediate and deep vascular plexus. Subsequently, the area was quantified using AngioTool64 (v0.6a) [30]. RPE cell morphology parameters (mean cell area, eccentricity, solidity, and cell number) of 30-week-old mice were analyzed in images of equal area (362 × 272 µM) using CellProfiler 4.0.7. Representative images were taken with the laser-scanning confocal Leica TCS SP8 (Leica Microsystems, Wetzlar, Germany).

### 2.4. RT-qPCR

RNA was isolated from retinas using the NucleoSpin^®^ RNA Kit (Macherey-Nagel, Düren, Germany) according to the manufacturer’s instructions. A measurement of 200 ng of total RNA was reverse transcribed with the RevertAid First Strand cDNA Synthesis Kit (Thermo Fisher Scientific, Waltham, MA, USA). Then, cDNA was diluted 1:5 in distilled water and used for real-time quantitative PCR (RT-qPCR) with PowerUp™ SYBR™ Green Master Mix (Thermo Fisher Scientific) on a QuantStudio™ 5 Real-Time PCR System (Thermo Fisher Scientific). Exon-spanning primers were designed using the UCSC Genome Browser to avoid genomic amplification. Primer specificity was verified by melting curve analysis and gel electrophoresis of PCR products. Primer sequences were as follows: *Fgf2* 5′-CTCCAGTTGGTATGTGGCACT-3′ (forward), 5′-TCAGCTCTTAGCAGACATTGGA-3′ (reverse); *Fgf5* 5′-AACTCCTCGTATTCCTACAATCC-3′ (forward), 5′-CGGATGGCAAAGTCAATGG-3′ (reverse); *Fgf21* 5′-GGGATGGGTCAGGTTCAGA-3′ (forward), 5′-CAGCCTTAGTGTCTTCTCAGC-3′ (reverse); *Vegfa* 5′-AGGGTCAAAAACGAAAGCGC-3′ (forward), 5′-CGCGAGTCTGTGTTTTTGCA-3′ (reverse); *Vegfb* 5′-AGAGTGCTGTGAAGCCAGAC-3′ (forward), 5′-CTGGGTTGAGCTCTAAGCCC-3′ (reverse); *Vegfc* 5′-TGCCGGTGCATGTCTAAACT-3′ (forward), 5′-TTAGCTGCCTGACACTGTGG-3′ (reverse); *Vegfed* 5′-TTCAGGAGCGAACATGGACC-3′ (forward), 5′-CCACAGCTTCCAGTCCTCAG-3′ (reverse).

### 2.5. Immunoblot

Retinas were homogenized using M-PER Mammalian Protein Extraction Reagent (Thermo Fisher Scientific, Waltham, MA, USA #78503) containing protease inhibitor (Sigma #11697498001) and Phosphatase Inhibitor Cocktail (Cell Signaling, Danvers, MA, USA #5870) with a Branson Sonifier W-450D at 40% amplitude. Proteins (30 μg per sample) were separated by a gradient SDS-PAGE (stacking 4%; intermediate 10%, separation 15%) at 80 V and transferred to a 0.45 μm polyvinylidene difluoride (PVDF) membrane for 90 min at 100 V. Membranes were blocked in 5% non-fat dry milk in Tris-buffered saline with Tween^®^20 (TBS-T) for 1 h at room temperature. Primary antibodies against FGF2 (1:2000; Merck Darmstadt, Germany #05-118) and GARP (1:1000; Sigma Burlington, MA, USA #MABN2429) were incubated in 5% non-fat dry milk overnight at 4 °C. Membranes were washed and incubated with corresponding HRP secondary antibody anti-mouse HRP (1:2000; Santa Cruz Biotechnology, Dallas, Texas, USA #sc-516102) for 1 h at room temperature. As loading contol, β-Actin-Peroxidase (1:6000, Sigma Burlington, MA, USA #A3854-200UL) was used. Proteins were detected using SuperSignal West Atto Ultimate Sensitivity substrate (Thermo Fisher Scientific, Waltham, MA, USA #A45917) and imaged using a Bio-Rad ChemiDoc MP (Bio-Rad, Hercules, CA, USA) and a Vilber Lourmat Fusion FX Edge imager (Vilber Lourmat, Eberhardzell, Germany).

### 2.6. TUNEL Assay

Apoptotic cell death was detected by the terminal deoxynucleotidyl transferase-mediated biotinylated UTP nick end labeling (TUNEL) assay using a commercial kit (In Situ Cell Death Detection Kit, Fluorescein; Roche, Basel, Switzerland #11684795910). The TUNEL assay was performed according to the manufacturer’s instructions on retinal cryosections. Sections treated with 60 U/mL DNase I were used as a positive control. Slides were then washed for 15 min in PBS and counterstained with 5 µg/mL Hoechst 33342, as described in Section 2.2.

### 2.7. Statistics

All data were analyzed and plotted with GraphPad Prism 9.3. Values are presented as mean ± SEM. Statistical comparisons were performed using ANOVA followed by Tukey’s post hoc test for multiple comparisons or Student’s *t*-test for pairwise comparison. *p* < 0.05 was considered statistically significant (*p* < 0.05; * *p* < 0.01; ** *p* < 0.001). Sample size (*n*) indicates the number of animals per genotype, as specified in figure legends.

## 3. Results

### 3.1. Loss of FGF2 Does Not Affect Müller Glia, Photoreceptors, or Retinal Vasculature

**Figure 1:**
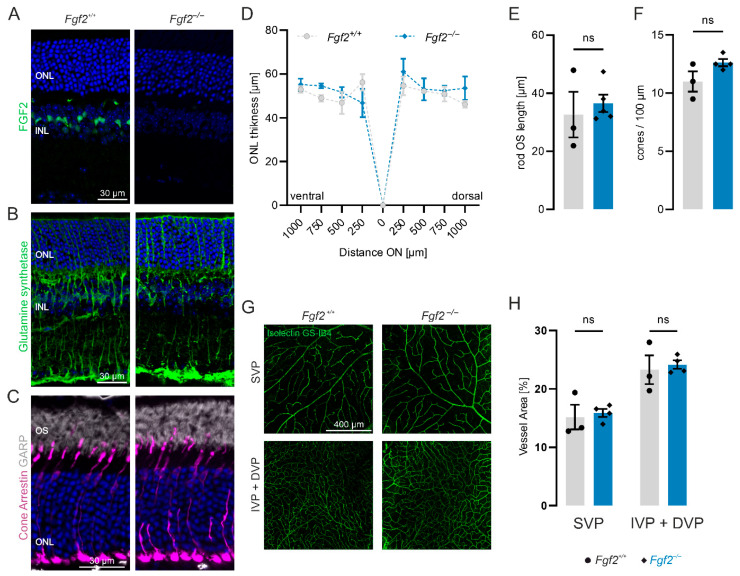
Loss of FGF2 does not affect the overall retinal structure or vascular network. Retinas from *Fgf2*^+/+^ (wild-type, grey) and *Fgf2*^−/−^ (blue) were analyzed. (**A**–**C**) Representative retinal cryosections immunostained for (**A**) FGF2 in 8-week-old mice, (**B**) glutamine synthetase (Müller glia) in 52-week-old mice, and (**C**) GARP (rod photoreceptor outer segments) and cone arrestin (cone photoreceptors) in 52-week-old mice. (**D**–**F**) Quantification of outer nuclear layer (ONL) thickness (**D**), rod outer segment (OS) length (**E**), and cone number (**F**) in 52-week-old wild-type and *Fgf2*^−/−^ mice. (**G**) Retinal flatmounts stained with isolectin GS-IB4. Maximum intensity projections of the superficial vascular plexus (SVP) and merged intermediate and deep plexuses (IVP+DVP) are shown. (**H**) Quantification of vessel area. Data are presented as mean ± SEM and were analyzed using Student’s *t*-test for pairwise comparison; *p* < 0.05 was considered statistically significant. ns, not significant. Each symbol represents one retina from an individual animal. The spider plot depicts mean ± SEM (*n* = 3 for wild type, *n* = 4 for *Fgf2*^−/−^).

To determine whether FGF2 is necessary for maintaining retinal integrity, we analyzed *Fgf2*-deficient (*Fgf2*^−/−^) mice in which exon 1 of the *Fgf2* gene was replaced with a neomycin cassette [28]. Immunolabeling of retinal cryosections confirmed FGF2 expression in wild-type retinas (Figure 1A), where FGF2 localized to the somata of Müller glial cells identified by glutamine synthetase (Supplementary Figure S1A). In *Fgf2*^−/−^ retinas, FGF2 was undetectable. Despite the absence of FGF2, Müller glia cells appeared morphologically normal (Figure 1B, Supplementary Figure S1B). Since Müller cells are a source of FGF2 and may release it to support retinal cells, we examined microglia, rods, and cones. The number of microglia was similar to that of wild type in *Fgf2*^−/−^ mice (Supplementary Figure S1C). Rod outer segments and cones were visualized using immunostaining for N-terminal glutamic-acid-rich protein (GARP) and cone arrestin, respectively (Figure 1C). Both rod outer segments and cones exhibited normal morphology in *Fgf2*^−/−^ retinas compared to wild type. To assess variation in retinal structure parameters quantitatively, we measured outer nuclear layer (ONL) thickness, rod outer segment length, and cone number in 52-week-old wild-type and *Fgf2*^−/−^ mice (Figure 1D–F). ONL thickness, measured at multiple positions, showed no significant difference between genotypes (Figure 1D). Similarly, rod outer segment length (Figure 1E) and cone number (Figure 1F) revealed no significant differences between wild-type and *Fgf2*^−/−^ retinas, indicating a preserved photoreceptor structure. In addition, cell death was not detected by TUNEL staining (Supplementary Figure S2). Since FGF2 is a pro-angiogenic factor [31], we also investigated whether FGF2 deficiency influences the vascular architecture. The mouse retinal vasculature forms a trilaminar network of superficial, intermediate, and deep vascular plexuses. Flatmounted retinas from 20-week-old wild-type and *Fgf2*^−/−^ mice were immunolabeled with isolectin GS-IB4, a marker for endothelial cells. All three plexuses appeared morphologically normal, with no evidence of vessel remodeling, angiogenesis, or degeneration in *Fgf2*^−/−^ retinas (Figure 1G). Quantifications of the vessel area in the superficial vascular plexus as well as in the deep and intermediate vascular plexuses confirmed the absence of significant vascular alterations (Figure 1H). Together, these findings demonstrate that FGF2 is dispensable for maintaining retinal architecture, photoreceptor integrity, Müller glia morphology, and vascular organization.

### 3.2. FGF2 Is Upregulated in Pde6b^STOP/STOP^ Retinas but Is Not Required for Photoreceptor Survival

**Figure 2:**
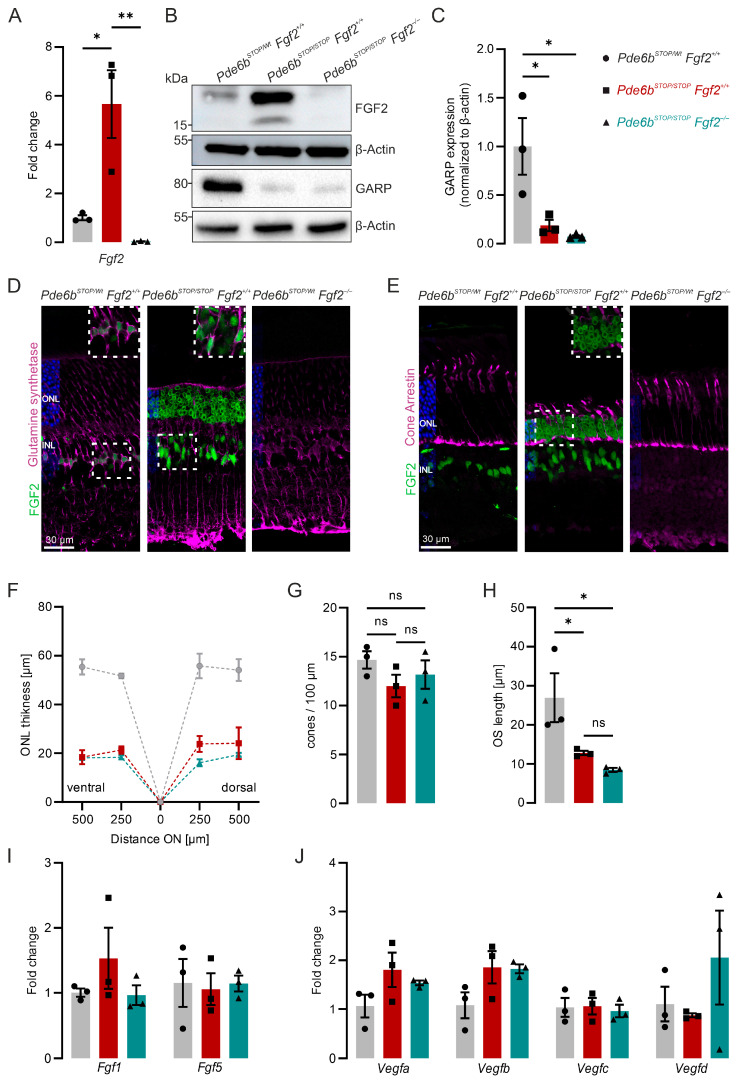
Absence of FGF2 in the degenerating retina does not exacerbate disease progression. Retinas from 8-week-old *Pde6b^STOP/Wt^ Fgf2*^+/+^ (gray), *Pde6b^STOP/STOP^ Fgf2*^+/+^ (red), and *Pde6b^STOP/STOP^ Fgf2*^−/−^ (teal) were analyzed. (**A**) RT-qPCR analysis of *Fgf2* transcripts. (**B**) Representative immunoblot of FGF2 and GARP from retinal lysates. β-Actin was used as a loading control. (**C**) Quantitative analysis of GARP immunoblots. (**D**,**E**) Representative retinal cryosections immunostained for FGF2 and glutamine synthetase (Müller glia) (**D**), and FGF2 and cone arrestin (cone photoreceptors) (**E**). Dashed boxes indicate a zoomed-in area. (**F**–**H**) Quantification of outer nuclear layer (ONL) thickness (**F**), cones (**G**), and rod outer segment (OS) length (**H**). (**I**,**J**) RT-qPCR analysis of *Fgf* family member transcripts (**I**) and *Vegf* family member transcripts (**J**). Data are presented as mean ± SEM and were analyzed using one-way ANOVA followed by Tukey’s post hoc test for multiple comparisons. ns, not significant, *p* < 0.05: *, *p* < 0.01: **. Each symbol represents one retina from an individual animal. The spider plot depicts mean ± SEM (*n* = 3 for *Pde6b^STOP/Wt^ Fgf2*^+/+^, *n* = 3 for *Pde6b^STOP/STOP^ Fgf2*^+/+^ and *n* = 3 for *Pde6b^STOP/STOP^ Fgf2*^−/−^).

FGF2 is upregulated in several mouse models of photoreceptor degeneration [13]. To examine the significance of this upregulation, we used *Pde6b^STOP/STOP^* mice, which carry a floxed STOP cassette in the *Pde6b* gene, preventing PDE6B expression and leading to progressive photoreceptor degeneration [32]. To assess whether FGF2 is also increased in this model, we analyzed FGF2 expression using RT-qPCR, immunoblot, and immunohistochemistry. At 8 weeks of age, *Ffg2* mRNA and FGF2 protein were significantly upregulated in *Pde6b^STOP/STOP^* retinas (Figure 2A,B). To better understand the role of FGF2 in RP, we crossed *Fgf2^−/−^* mice with *Pde6b^STOP/STOP^* mice to derive *Pde6b^STOP/STOP^ Fgf2*^−/−^ mice (vs. *Pde6b^STOP/Wt^ Fgf2*^+/+^ and *Pde6b^STOP/STOP^ Fgf2*^+/+^ control mice). Neither *Ffg2* mRNA nor FGF2 protein was detected in these mice (Figure 2A,B). Immunoblotting for rod photoreceptor outer segments revealed that GARP expression was significantly decreased in both 8-week-old *Pde6b^STOP/STOP^* and *Pde6b^STOP/STOP^ Fgf2*^−/−^ retinas compared to age-matched *Pde6b^STOP/WT^* retinas, with no significant difference between the mutant groups (Figure 2B,C). Immunolabeling revealed that FGF2 is expressed not only in Müller glial cells (Figure 2D) but also in photoreceptor cell bodies in *Pde6b^STOP/STOP^* retinas (Figure 2D,E). The lack of co-localization between FGF2 and cone arrestin-positive cells suggests that FGF2 is expressed in rod photoreceptors, indicating that photoreceptor degeneration triggers the FGF2 response in rods. Next, we analyzed retinal cryosections at 8 weeks of age. The 8-week time point was chosen because it represents a well-established stage of advanced photoreceptor degeneration in the *Pde6b^STOP/STOP^* model, where ONL thinning is clearly detectable while the residual structure still allows comparative analysis [32]. ONL thickness (Figure 2F), rod OS length (Figure 2G), and cone number (Figure 2H) were measured. None of these morphometric features differed significantly between *Pde6b^STOP/STOP^ Fgf2*^+/+^ and *Pde6b^STOP/STOP^ Fgf2*^−/−^ mice, suggesting that FGF2 deficiency does not accelerate photoreceptor degeneration. To test potential compensatory changes, we measured the expression of *Fgf1*, *Fgf5*, *Vegfa*, *Vegfb*, *Vegfc*, and *Vegfd* by RT-qPCR. No upregulation of these genes was detected in *Pde6b^STOP/STOP^ Fgf2*^−/−^ retinas (Figure 2I,J). Further, we analyzed VEGFA expression in retinal sections (Supplementary Figure S3). In line with the RT-qPCR data, the expression level did not differ between genotypes. Together, these results indicate that although FGF2 is upregulated in degenerating retinas and is expressed in both Müller glia and rod photoreceptors, its absence does not exacerbate photoreceptor loss.

### 3.3. FGF2 Deficiency Has No Effect on RPE Morphological Changes During Retinal Degeneration

**Figure 3:**
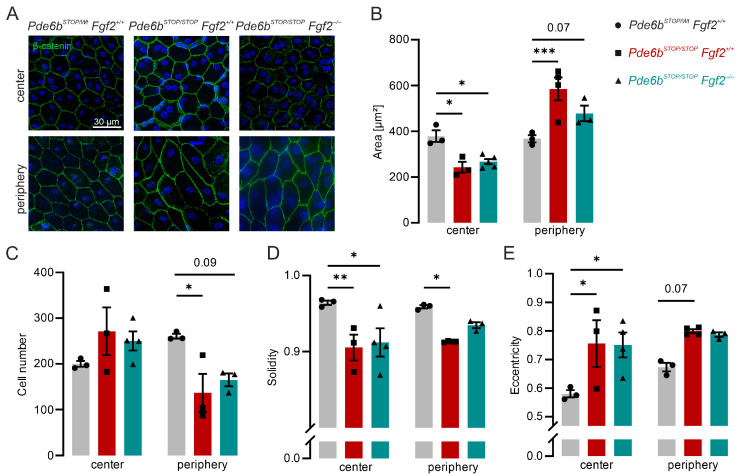
Loss of FGF2 does not affect morphological changes in the RPE in the degenerating retina. RPE–choroid–sclera flatmounts from 30-week-old *Pde6b^STOP/Wt^ Fgf2*^+/+^ (gray), *Pde6b^STOP/STOP^ Fgf2*^+/+^ (red), and *Pde6b^STOP/STOP^ Fgf2*^−/−^ (teal) mice were analyzed. (**A**) Representative images of RPE–choroid flatmounts immunolabeled for β-catenin. (**B**–**E**) Quantification of RPE cell area (**B**), RPE cell number covering an area of 362 × 272 µM (**C**), solidity (**D**), and eccentricity (**E**). Data are presented as mean ± SEM and were analyzed using one-way ANOVA followed by Tukey’s post hoc test for multiple comparisons. *p* < 0.05: *, *p*< 0.01: **, *p* < 0.001: ***. Each symbol represents one RPE from an individual animal.

The RPE plays a critical role in maintaining photoreceptor homeostasis [33]. To investigate whether FGF2 influences RPE integrity during degeneration, we examined RPE–choroid–sclera flatmounts from 30-week-old mice. Preparations were immunolabeled with β-catenin, a component of the adherens junction complex, to visualize RPE cell borders (Figure 3A). Because RPE remodeling can differ between central and peripheral regions [32], both regions were analyzed separately. In *Pde6b^STOP/Wt^ Fgf2*^+/+^ control mice, RPE cells displayed a uniform, mostly hexagonal, morphology characteristic of healthy RPE. By contrast, RPE cells from both *Pde6b^STOP/STOP^ Fgf2*^+/+^ and *Pde6b^STOP/STOP^ Fgf2*^−/−^ mice exhibited structural alterations. In the central retina, cells lost their regular hexagonal organization and appeared polymorphic, with very small cells. In the periphery, cells were elongated and irregularly aligned, reflecting a pronounced remodeling response. To quantify these changes, we measured RPE cell area, cell number, solidity, and eccentricity. In the central RPE, the mean cell area was significantly decreased in both *Pde6b^STOP/STOP^ Fgf2*^+/+^ and *Pde6b^STOP/STOP^ Fgf2*^−/−^ compared to *Pde6b^STOP/Wt^ Fgf2*^+/+^ control. No significant differences were observed between *Pde6b^STOP/STOP^ Fgf2*^+/+^ and *Pde6b^STOP/STOP^ Fgf2*^−/−^ mice. In the periphery, the RPE area was increased relative to the control (Figure 3B). Cell numbers were slightly, but not significantly, increased in the central region and decreased in the periphery in both *Pde6b^STOP/STOP^* and *Pde6b^STOP/STOP^ Fgf2*^+/+^ mice compared to *Pde6b^STOP/Wt^* controls. These changes correspond to regional differences in cell area (Figure 3B). Solidity reflects the proportion of the cell area enclosed by a best-fit convex envelope. In the central retina, both mutants exhibited significantly lower solidity relative to the control. In the periphery, however, only *Pde6b^STOP/STOP^* mice showed significant reductions in solidity, whereas *Pde6b^STOP/STOP^ Fgf2*^−/−^ did not differ significantly from *Pde6b^STOP/Wt^ Fgf2*^+/+^ control (Figure 3D). Eccentricity, a measure of cell elongation, was significantly elevated in the central retina of both *Pde6b^STOP/STOP^ Fgf2*^+/+^ and *Pde6b^STOP/STOP^ Fgf2*^−/−^ mice compared with *Pde6b^STOP/Wt^ Fgf2*^+/+^ control, indicating decreased deformation of the cell shape (Figure 3E). Investigations of tight junctions stained with ZO-1 were disrupted in both *Pde6b^STOP/STOP^ Fgf2*^+/+^ and *Pde6b^STOP/STOP^ Fgf2*^−/−^ compared to *Pde6b^STOP/Wt^ Fgf2*^+/+^ control (Supplementary Figure S4). These results indicate that loss of FGF2 does not exacerbate RPE morphological changes in *Pde6b^STOP/STOP^* mice.

### 3.4. Loss of FGF2 Enhances Microglia During Early Photoreceptor Degeneration

**Figure 4:**
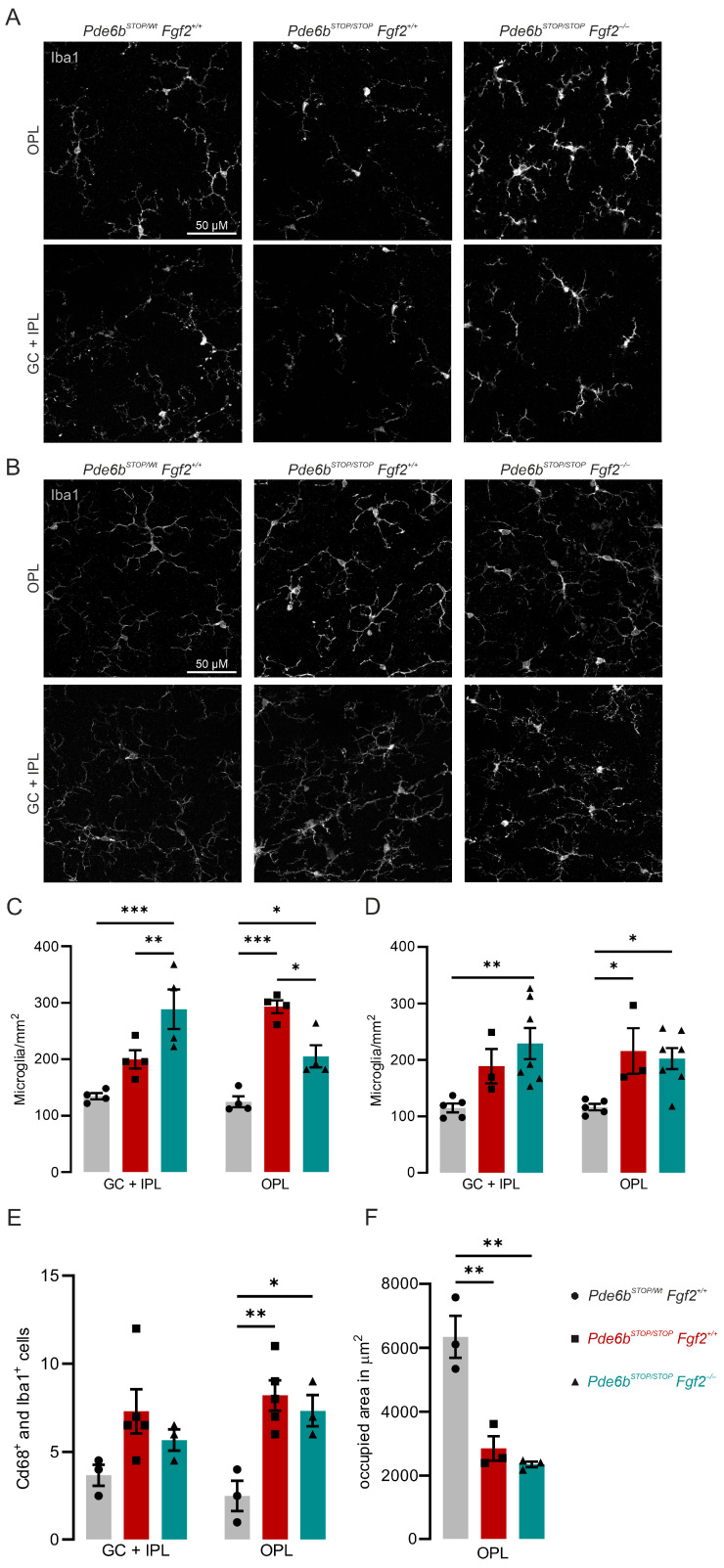
Loss of FGF2 leads to an early microglial activation in the degenerating retina. Retinas from *Pde6b^STOP/Wt^ Fgf2*^+/+^ (gray), *Pde6b^STOP/STOP^ Fgf2*^+/+^ (red), and *Pde6b^STOP/STOP^ Fgf2*^−/−^ (teal) mice were analyzed. (**A**,**B**) Representative flatmounted retinas immunostained for Iba1 at 8 weeks of age (**A**) and 30 weeks of age (**B**). (**C**,**D**) Quantification of Iba1-positive microglia at 8 weeks of age (**C**) and 30 weeks of age (**D**). Quantification of Cd68-positive and Iba1-positive cells in retinal sections (8-week-old mice) (**E**). Quantification of the occupied area by microglia in the OPL from flatmounted retinas (8-week-old mice) (**F**). Data are presented as mean ± SEM and analyzed by one-way ANOVA followed by Tukey post hoc test for multiple comparisons. *p* < 0.05: *, *p* < 0.01: **, *p* < 0.001: ***. Each symbol represents one retina from one individual. GC + IPL, ganglion cell plus inner plexiform layer; OPL, outer plexiform layer.

Degenerating neurons release diverse signaling molecules, including FGF2, that act on surrounding glial cells and have been classified as ‘find-me’, ‘help-me’, and ‘eat-me’ signals [34,35]. In RP patients and mouse models, microglia respond to rod degeneration by becoming reactive, adopting an activated morphology, and migrating into the photoreceptor layer [36,37]. To determine whether FGF2 influences this process, we performed Iba1 immunolabeling on retinal flatmounts at 8 and 30 weeks of age (Figure 4A,B). In 8-week-old control *Pde6b^STOP/Wt^ Fgf2*^+/+^ retinas, microglia exhibited a quiescent morphology characterized by a regular, non-overlapping mosaic of evenly spaced cells with small somata and fine, highly branched processes. In *Pde6b^STOP/STOP^ Fgf2*^+/+^ retinas, microglia appeared denser, with early signs of activation, including process retraction and thickening. This effect was further enhanced in *Pde6b^STOP/STOP^ Fgf2*^−/−^ retinas, where microglia displayed a markedly amoeboid morphology, with shortened, thickened processes and enlarged somata, consistent with a highly activated state. To quantify microglial migration, we counted Iba1-positive cells in the ganglion cell plus inner plexiform layer (GC + IPL) and in the outer plexiform layer (OPL).

At 8 weeks, *Pde6b^STOP/STOP^ Fgf2*^+/+^ retinas exhibited a significant increase in microglial cell numbers in the OPL compared with *Pde6b^STOP/Wt^ Fgf2*^+/+^ controls. In contrast, *Pde6b^STOP/STOP^ Fgf2*^−/−^ retinas showed a marked increase in microglial density within the GC + IPL relative to both *Pde6b^STOP/Wt^ Fgf2*^+/+^ and *Pde6b^STOP/STOP^ Fgf2*^+/+^ retinas. In the OPL, microglial numbers in *Pde6b^STOP/STOP^ Fgf2*^−/−^ retinas were also significantly elevated compared with controls, but remained significantly lower than those observed in *Pde6b^STOP/STOP^ Fgf2*^+/+^ retinas (Figure 4C). These findings suggest that FGF2 deficiency alters microglial activation and migration in the early stages of degeneration. To test whether these effects persist during later disease stages, we examined microglia at 30 weeks of age. At this age, both *Pde6b^STOP/STOP^ Fgf2*^+/+^ and *Pde6b^STOP/STOP^ Fgf2*^−/−^ retinas exhibited a marked increase in microglial density, but no significant differences between genotypes were observed (Figure 4D). We next analyzed Cd68-positive microglia cells, which were upregulated in the OPL of both *Pde6b^STOP/STOP^ Fgf2*^+/+^ and *Pde6b^STOP/STOP^ Fgf2*^−/−^ retinas (Figure 4E, Supplementary Figure S5). In 8-week-old mutant retinas, the microglial-occupied area in the OPL was significantly decreased compared to wild-type control (Figure 4F). Thus, the influence of FGF2 on microglial migration appears to be most pronounced during the early phase of photoreceptor degeneration and diminishes as degeneration progresses. Taken together, these results indicate that FGF2 normally enhances microglial migration in the OPL, thereby potentially modulating the local inflammatory response.

## 4. Discussion

FGF2 is suggested to act as a survival factor for various types of neurons, including photoreceptor cells [20,38,39]. Upregulation of FGF2 has been shown to reduce cell death induced by diverse insults, including ischemia, neurotoxic exposure, excitotoxicity, and nitric oxide [40,41,42,43,44,45]. In this study, we examined the role of FGF2 in maintaining retinal integrity and its influence on retinal degeneration. Using *Fgf2*^−/−^ mice, we demonstrated that the loss of Fgf2 does not affect Müller glia, which normally express FGF2 under healthy conditions, nor does it alter photoreceptor or vasculature development. These data are consistent with a previous study showing that 17-day-old *Fgf2*-deficient mice have normal retinal blood vessels and retinal structure [46]. On the other hand, FGF receptor signaling has been shown to be a critical regulator of vascular development. In the absence of FGF signaling, glycolysis was decreased, leading to impaired endothelial cell proliferation and migration [42]. Consistent with this concept, FGF2-driven signaling in retinal vascular pathologies has recently been linked to endothelial metabolic reprogramming and neovascularization [24]. The normal retinal structure (including blood vessels and RPE) in *Fgf2*^−/−^ mice could be due to compensatory effects, which may mask phenotypic effects. Moreover, because FGF2 deficiency persists throughout development, adaptive or compensatory changes may occur, complicating the interpretation of its role in adult retinal homeostasis and degeneration.

In the context of photoreceptor degeneration, FGF2 is highly upregulated [9,10,11,13]. Our results show that loss of FGF2 has neither a sustained protective nor deleterious effect on photoreceptor cells in the degenerating retina. In addition, the RPE, which is essential for maintaining photoreceptor homeostasis and overall retinal health, undergoes remodeling in response to photoreceptor degeneration [33]. Previous in vitro work demonstrated that exogenous FGF2 can reduce apoptosis in primary bovine RPE cells by activating survival pathways [47]. However, in the *Pde6b^STOP/STOP^* mouse model, the absence of FGF2 did not result in increased structural disorganization.

Because degenerating photoreceptors release stress signals, including FGF2, we next investigated whether FGF2 deficiency alters microglial reactivity during retinal degeneration. In the retina, microglia primarily reside in two distinct synaptic layers, the outer and inner plexiform layers (OPL and IPL, respectively). Photoreceptor degeneration triggers microglial activation, leading to their infiltration into the photoreceptor layer and phagocytosis of photoreceptor debris [48]. FGF2 has been proposed as an important signaling molecule mediating crosstalk between degenerating neurons and microglia [49]. Consistent with this notion, FGF2 administration in the injured rat brain elicits a robust glial response [50]. FGF2 has also been implicated in regulating immune cell behavior and anti-inflammatory and wound-healing effects [5,51,52,53]. In the early stages of disease in the *Pde6b^STOP/STOP^* mouse model, FGF2 deficiency transiently increased microglial numbers in the IPL while reducing them in the OPL. These findings indicate that FGF2 differentially modulates microglia numbers across the two synaptic layers at early stages of retinal degeneration. Given that microglia exhibit distinct properties depending on their anatomical location in the retina [54], our results suggest the spatiotemporal dependence of microglial engagement on FGF2 signaling. Since our analysis is primarily based on microglial distribution and morphology, without comprehensive molecular characterization of inflammatory signaling pathways, mechanistic studies that account for both temporal and structural aspects of neuron–microglia interactions are needed.

In summary, our results suggest that FGF2, while robustly upregulated in response to retinal stress, does not significantly alter photoreceptor survival. Since we did not perform functional assessments of retinal activity, such as electroretinography or behavioral tests, conclusions regarding the potential functional consequences of FGF2 deficiency are limited. Moreover, although we analyzed multiple time points, subtle or transient effects may have been missed, and the selection of representative stages may not fully capture dynamic disease processes. Future studies incorporating functional assays, molecular profiling, and conditional approaches will be important to address these limitations.

## Data Availability

The original contributions presented in this study are included in the article/Supplementary Materials. Further inquiries can be directed to the corresponding author.

## Supplementary Materials

The following supporting information can be downloaded at: https://www.mdpi.com/article/10.3390/cells15070643/s1, Supplementary Data S1.

## Abbreviations

The following abbreviations are used in this manuscript:

AAV	adeno-associated viruses
DVP	deep vascular plexus
FGF2	fibroblast growth factor 2
GARP	glutamic-acid-rich protein
GC + IPL	ganglion cell plus inner plexiform layer
IVP	intermediate vascular plexus
ONL	outer nuclear layer
OPL	outer plexiform layer
OS	outer segments
PVDF	polyvinylidene difluoride
RCS	Royal College of Surgeons
RP	retinitis pigmentosa
RPE	retinal pigment epithelium
RT-qPCR	real-time quantitative PCR
SVP	superficial vascular plexus
TBS-T	Tris-buffered saline with Tween^®^20
TUNEL	transferase-mediated biotinylated UTP nick end labeling
