# Supplementary material for: FGF2 Deficiency Modulates Early Microglial Responses Without Affecting Photoreceptor Survival in a Retinitis Pigmentosa Mouse Model
Source: Cells. 2026 Apr 2;15(7):643. doi: 10.3390/cells15070643 (PMC13073567; doi:10.3390/cells15070643)
Supplement: Supplementary file 1 [file cells-15-00643-s001.zip › cells-4155966-supplementary.pdf]

Supplementary Data S1

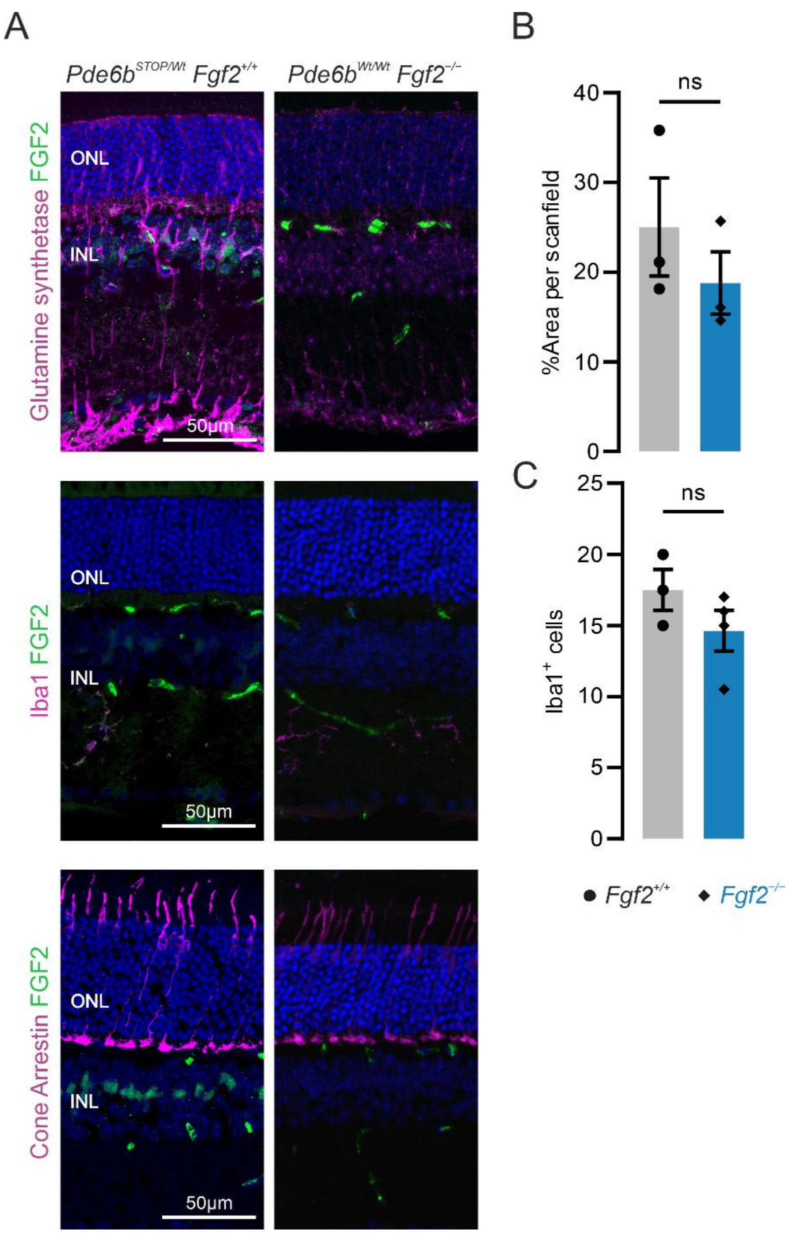

Supplementary Figure S1. **(A)** Representative images of *Pde6b*$^{STOP/Wt}$ *Fgf2*$^{+/+}$ (grey) and *Pde6b*$^{Wt/Wt}$ *Fgf2*$^{-/-}$ (blue) retinal cryosections immunostained for FGF2 and glutaminase synthetase (52-week-old mice), FGF2 and Iba1, as well as FGF2 and cone arrestin (cone photoreceptors) (8-week and 52-week-old animals, respectively). **(B-C)** Quantitative analysis of Müller glia **(B)** and Iba1-positive cell count **(C)** in *Pde6b*$^{STOP/Wt}$ *Fgf2*$^{+/+}$, *Pde6b*$^{Wt/Wt}$ *Fgf2*$^{-/-}$ retinal sections.

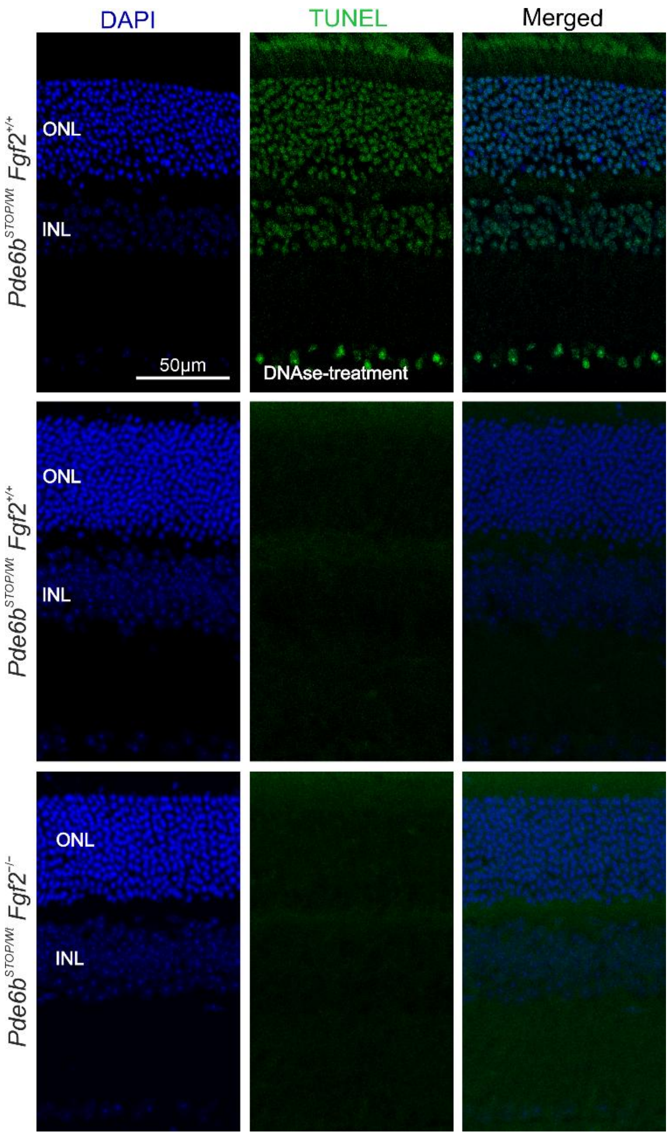

Supplementary Figure S2. Representative images of retinal sections stained for TUNEL from 8-week-old *Pde6b*$^{STOP/Wt}$ *Fgf2*$^{+/+}$, *Pde6b*$^{STOP/STOP}$ *Fgf2*$^{+/+}$, and *Pde6b*$^{STOP/STOP}$ *Fgf2*$^{-/-}$ mice.

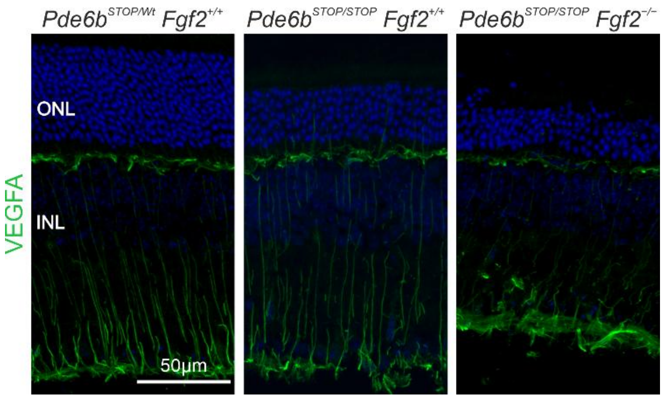

Supplementary Figure S3. Representative images of retinal cryosections immunostained for VEGFA from 8-week-old *Pde6b*$^{STOP/Wt}$ *Fgf2*$^{+/+}$, *Pde6b*$^{STOP/STOP}$ *Fgf2*$^{+/+}$, and *Pde6b*$^{STOP/STOP}$ *Fgf2*$^{-/-}$ mice.

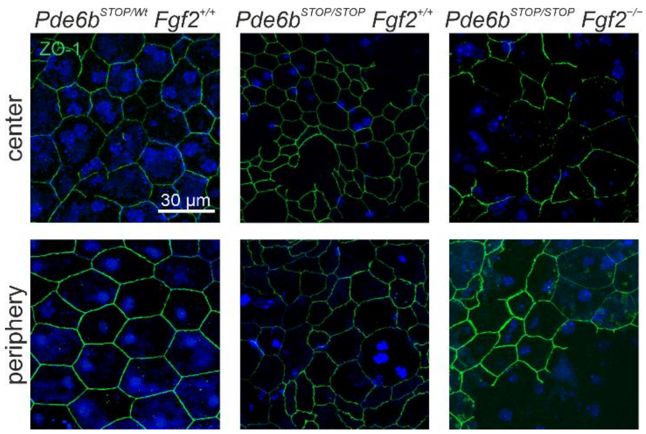

Supplementary Figure S4. Representative images of RPE-choroid-sclera flatmounts immunolabeled for ZO-1 from 52-week-old *Pde6b*$^{STOP/Wt}$ *Fgf2*$^{+/+}$, *Pde6b*$^{STOP/STOP}$ *Fgf2*$^{+/+}$, and *Pde6b*$^{STOP/STOP}$ *Fgf2*$^{-/-}$ mice.

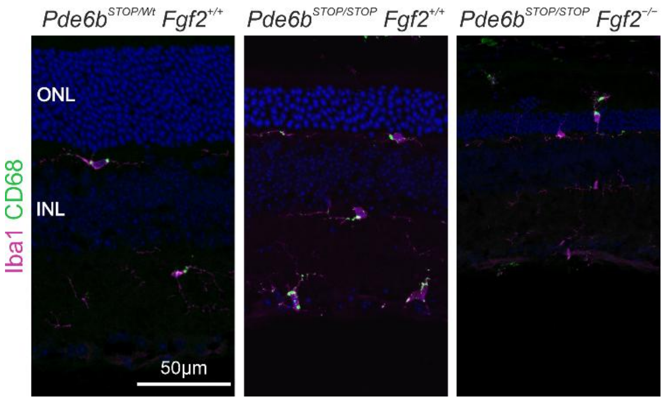

Supplementary Figure S5. Representative images of retinal cryosections immunostained for Iba1 and CD68 from 8-week old *Pde6b*$^{STOP/Wt}$ *Fgf2*$^{+/+}$, Pde6b$^{STOP/STOP}$ *Fgf2*$^{+/+}$ and *Pde6b*$^{STOP/STOP}$ *Fgf2*$^{-/-}$ mice.

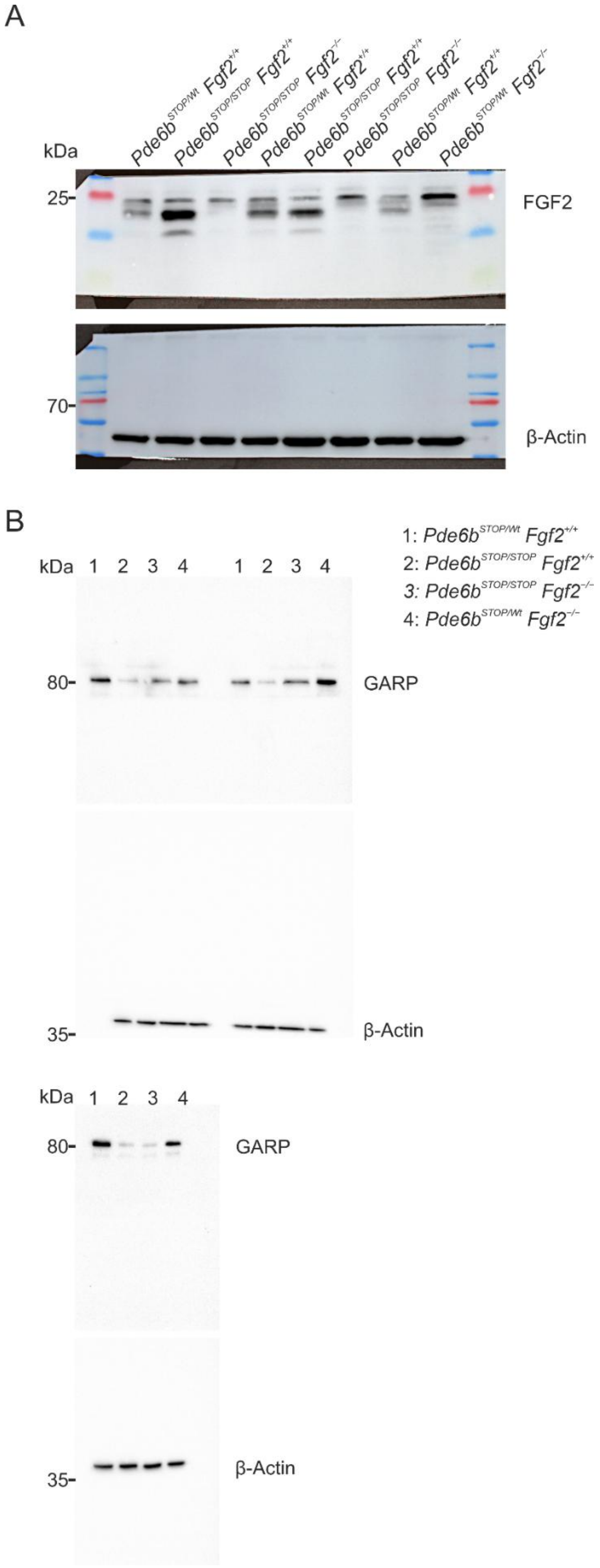

Supplementary Figure S6. (A) Uncropped immunoblots for FGF2 and β-Actin of retinal lysates from $Pde6b^{STOP/Wt}$ $Fgf2^{+/+}$, $Pde6b^{STOP/STOP}$ $Fgf2^{+/+}$, $Pde6b^{STOP/STOP}$ $Fgf2^{-/-}$, and $Pde6b^{STOP/Wt}$ $Fgf2^{-/-}$ mice. β-Actin was used as a loading control. **(B)** Uncropped immunoblots for GARP and β-Actin of retinal lysates $Pde6b^{STOP/Wt}$ $Fgf2^{+/+}$, $Pde6b^{STOP/STOP}$ $Fgf2^{+/+}$, and $Pde6b^{STOP/STOP}$ $Fgf2^{-/-}$ mice. β-Actin was used as a loading control. $Pde6b^{STOPt/Wt}$ $Fgf2^{-/-}$ was blotted as a control and not used for quantification purposes.
